# Overview of polymeric polyplexes for dsRNA delivery in insects: complexation, stability, and design considerations

**DOI:** 10.3389/finsc.2026.1750429

**Published:** 2026-01-21

**Authors:** Triin Kallavus, Jonathan Willow, Kristof De Schutter, Clauvis Nji Tizi Taning, Eve Veromann

**Affiliations:** 1 Chair of Plant Health, Institute of Agricultural and Environmental Sciences, Estonian University of Life Sciences, Tartu, Estonia; 2 Molecular Entomology Lab, Department of Plants and Crops, Faculty of Bioscience Engineering, Ghent University, Ghent, Belgium; 3 Pheasants Forever and Quail Forever, St. James, MN, United States

**Keywords:** insect, nanoparticle, pest management, polymeric polyplexes, RNAi

## Abstract

Polymer-based delivery systems for double-stranded RNA (dsRNA) have gained attention as a promising strategy for RNA interference (RNAi)-mediated insect pest control. Despite encouraging *in vitro* results, their practical application remains limited by methodological inconsistencies and species-specific challenges. Variability in experimental parameters, such as nitrogen/phosphorous (N/P) ratios, dsRNA lengths, and buffer systems, complicates reproducibility and cross-study comparisons. Moreover, *in vitro* stability and transfection efficiency often fail to predict *in vivo* RNAi outcomes, highlighting the need for more physiologically relevant models. Variation in RNAi efficiency across insect orders, such as Lepidoptera and Hemiptera, continues to challenge the generalizability of polymer-based delivery systems. To advance the field, future research should focus on species-tailored polymer design, improved predictive assays, and comprehensive environmental safety evaluations. Interdisciplinary collaboration will be essential to develop RNAi delivery platforms that are efficient, scalable, and ecologically sustainable.

## 1 Introduction

RNA interference (RNAi) has emerged as a powerful and highly specific tool for insect pest control. By silencing essential genes through the delivery of double-stranded RNA (dsRNA), RNAi provides a sustainable alternative to conventional insecticides, which frequently drive resistance evolution, threaten non-target organisms and contribute to environmental contamination (1–4). The species-specific mode of action and capacity to target pests previously considered untreatable make RNAi an attractive component of integrated pest management (IPM) strategies (5). However, the practical implementation of RNAi-based strategies in insect pest management is hindered by multiple biological and technical challenges. One of the major limitations is the rapid degradation of dsRNA in insect gut, particularly in species with highly alkaline midgut environments and abundant nucleases, such as Lepidoptera (6–8). In addition to cellular uptake limitations, the endosomal escape of internalized dsRNA represents a critical barrier for achieving efficient RNA interference (4). Even when uptake occurs, dsRNA may be retained and degraded within endosomes, and the systemic spread of the RNAi signal often remains inefficient, restricting gene silencing to localized tissues (9). To overcome these obstacles, researchers have explored various delivery systems, among which polycationic polymers have shown considerable promise. These complexes are formed by electrostatic complexation between cationic polymers and dsRNA, resulting in nanoparticles that can protect dsRNA from enzymatic degradation, facilitate cellular uptake, and enhance delivery efficiency (6, 9).

The physicochemical properties of polyplexes, such as particle size, polydispersity index (PDI), and zeta potential, are strongly influenced by the polymer type and the nitrogen-to-phosphate (N/P) ratio used during complexation. These parameters determine the structural integrity, cellular uptake efficiency, and biological activity of the polyplexes (10). For example, near the isoelectric point, polyplexes tend to aggregate and form larger particles, while at optimal N/P ratios, they remain small and stable (11). Polymers with cationic and hydrophobic properties generally enhance dsRNA stability and cellular uptake (11). In particular, guanidine-containing polymers, whose hydrophobicity contributes to improved interaction with insect midgut cells, have demonstrated enhanced protection of dsRNA against nucleolytic degradation and more efficient internalization (6). Optimizing these parameters is therefore crucial for achieving stable and effective delivery systems in insect pest control (12).

Recent studies have demonstrated that polyplexes formed at optimized N/P ratios can exhibit favorable characteristics, such as small particle size, reduced surface charge, and improved RNA protection and transfection efficiency (13, 14). These physicochemical properties are critical to ensure efficient cellular uptake and minimize premature degradation. However, increasing polymer concentration beyond optimal levels may lead to non-specific toxicity, primarily due to the high density of positive charges in polycationic carriers, which can destabilize cellular membranes (6, 12). To mitigate this, chemical modifications such as the incorporation of guanidine groups—known for their ability to distribute charge more evenly—have been shown to reduce membrane disruption and improve biocompatibility.

While polymer-based RNAi delivery has demonstrated promising outcomes in certain some insect taxa, like coleoptera, numerous studies report limited or inconsistent gene knockdown in other taxa, like lepidopteran pests (6, 15, 16). These variable results underscore that delivery success is highly context-dependent and that polymer design and administration strategies must be carefully tailored to the physiological and cellular characteristics of each target species. Recognizing and addressing these limitations is critical for developing broadly effective RNAi platforms for pest management.

This review focuses on the complexation of dsRNA with polycationic polymers, the stability of resulting polyplexes, and the criteria for selecting suitable nanocarriers for RNAi delivery in insect systems. By synthesizing current knowledge, we aim to provide a conceptual framework for the rational design of RNAi-based pest control strategies that are tailored to the unique physiological barriers of insect pests.

## 2 Design and evaluation of polymeric nanocarriers for insect RNAi delivery

### 2.1 Polymeric nanocarriers in insect RNAi

Polymers can play a pivotal role in the delivery of dsRNA, the primary active molecule used in RNAi-based pest control strategies. By forming protective complexes with dsRNA, polymers shield it from enzymatic degradation in both environmental and biological contexts, while also promoting cellular uptake and intracellular trafficking (17, 18). The inherent instability of unformulated (‘naked’) dsRNA – arising from factors such as ultraviolet (UV) radiation, microbial activity, and nuclease degradation, has driven the development of polymer-based nanocarriers designed to enhance its stability and bioavailability (9, 18).

Both synthetic and natural polymers have been explored for dsRNA delivery. Synthetic polymers, such as poly(ethyleneimine) (PEI), poly(2-aminoethyl methacrylate) (PAEMA), and poly(glycidyl methacrylate) (PGUMA), offer tunable chemical properties, including adjustable charge density and molecular weight, which are critical for optimizing dsRNA binding and cellular uptake (9, 19, 20). Natural polymers like chitosan are valued for their biocompatibility, biodegradability, and low toxicity, making them attractive for environmentally friendly applications (21–23).

Poly(ethyleneimine) (PEI) is one of the most extensively studied synthetic polymers for nucleic acid delivery, including dsRNA. Its high cationic charge density, stemming from abundant primary, secondary, and tertiary amine groups, enables strong electrostatic interactions with the negatively charged phosphate backbone of nucleic acids (24–26). This property allows PEI to condense dsRNA into stable polyplexes that protect it from enzymatic degradation and facilitate cellular uptake via endocytosis (27). PEI is available in both linear and branched forms, with molecular weights ranging from a few hundred Daltons (Da) to several hundred kilodaltons (kDa) (28, 29). Among these, branched PEI with a molecular weight of 25 kDa is commonly used due to its high transfection efficiency (30). However, this efficiency comes at the cost of increased cytotoxicity, which is directly correlated with molecular weight and charge density (24, 31). Lower molecular weight variants (e.g., 1.8 kDa) offer improved biocompatibility but reduced delivery efficiency (32). This limitation can be addressed by blending strategies, which involve mixing different polymers to combine their advantageous properties, or by chemical modifications that enhance cellular uptake or stability. The effects of polymer properties on dsRNA binding and uptake are detailed in **Table 1**.

**Table 1:** The effectiveness of polymers in dsRNA delivery is primarily governed by three key chemical properties.

Property	Positive effect	Negative effect
Charge density	High cationic charge enhances electrostatic binding to dsRNA, promoting stable complex formation.	Excessive charge can increase cytotoxicity
Molecular weight	Higher molecular weight polymers can improve dsRNA protection and facilitate cellular uptake.	May reduce solubility and increase viscosity, potentially hindering formulation and delivery efficiency.
Hydrophilicity	Hydrophilic polymers are more soluble and biocompatible, supporting interactions with biological membranes.	Excessive hydrophilicity may limit membrane penetration and intracellular delivery, especially via non-endocytic pathways.

To address the cytotoxicity and improve biocompatibility, PEI can be chemically modified. One common approach is PEGylation, where polyethylene glycol (PEG) chains are grafted onto the PEI backbone (33–35). This reduces surface charge, minimizes non-specific interactions, and enhances solubility and circulation time in biological systems (33, 35). Other modifications include acetylation, carboxylation, and hydroxylation, all aimed at shielding the positive charges and reducing membrane destabilization (35). PEI’s “proton sponge” effect, its ability to buffer endosomal pH, also contributes to efficient endosomal escape, a critical step in intracellular delivery of dsRNA (27). This mechanism allows PEI-based complexes to release their cargo into the cytoplasm, enhancing gene silencing efficacy. Despite its limitations, PEI remains a cornerstone material in non-viral gene and RNA delivery systems due to its tunability, strong nucleic acid binding, and versatile chemical reactivity (24, 35).

Poly(2-aminoethyl methacrylate) (PAEMA) and Poly(glycidyl methacrylate) (PGUMA) are methacrylate-based polymers that also offer highly tunable chemical properties, making them attractive candidates for nucleic acid delivery systems, including dsRNA (9, 36). PAEMA is a cationic polymer formed from the monomer 2-aminoethyl methacrylate, and its structure includes primary amine groups, which confer a high positive charge density. This enables strong electrostatic interactions with the negatively charged phosphate backbone of dsRNA, facilitating efficient complexation and protection against enzymatic degradation. PGUMA, on the other hand, contains reactive epoxide side chains, which allow for post-polymerization functionalization through nucleophilic ring-opening reactions (37). These reactions enable the incorporation of various functional groups, such as amines, thiols, azides, and acids, allowing for the addition of targeting ligands, stabilizing agents, or responsive moieties (38). This versatility makes PGUMA a highly adaptable scaffold for designing delivery systems tailored to specific biological environments or therapeutic goals. Both PAEMA and PGUMA are hydrophilic, which enhances their solubility and biocompatibility (39). Moreover, they can be synthesized with controlled molecular weights, allowing researchers to fine-tune their physical properties, such as particle size, charge density, and degradation rate, to optimize delivery performance (36). Recent studies have demonstrated that PAEMA derivatives, such as phenylboronic acid-functionalized PAEMA, can effectively bind siRNA due to their high-density positive charges, and facilitate cellular uptake and gene silencing (40, 41). Similarly, PGUMA-based systems have been used to create multifunctional nanoparticles for drug and gene delivery, benefiting from the polymer’s chemical reactivity and structural stability (42).

Chitosan, a natural cationic polysaccharide derived from chitin—the second most abundant biopolymer, is biodegradable, biocompatible, and low in toxicity, making it attractive for field applications. Its charge density, influenced by the degree of deacetylation, enhances interactions with negatively charged molecules such as dsRNA and siRNA. Chitosan is typically obtained from crustacean shells, a by-product of seafood processing, adding to its sustainability (43, 44). The property significantly improves its ability to form stable complexes for gene silencing purposes. Chitosan is also highly hydrophilic, which supports its solubility in aqueous environments and facilitates interactions with biological membranes (45). These characteristics make chitosan suitable for both mucosal and systemic delivery routes, as it can traverse epithelial barriers and maintain bioactivity under a range of physiological conditions.

In addition to widely studied carriers such as chitosan and guanidine-modified polymers, recent advances have introduced a broader spectrum of polymeric and nanoparticle-based systems for RNAi delivery in insect models. Notably, Layered Double Hydroxide (LDH) nanoparticles, also known as Bioclay, are promising carriers for dsRNA delivery due to their positively charged layered structure, which enables strong electrostatic interaction with negatively charged nucleic acids what have shown significant promise in enhancing dsRNA stability and systemic transport in plants (46–48). These carriers facilitate adhesion to leaf surfaces, internalization into plant cells, and translocation through vascular tissues, ultimately improving RNAi efficacy against piercing-sucking pests such as *Aphis gossypii* Glover (48).

Another innovative class includes Star Polycations (SPc), which are highly branched polycationic nanocarriers capable of forming stable complexes with dsRNA through electrostatic and hydrogen bonding interactions (9, 49). SPc-based systems have demonstrated enhanced protection of dsRNA against degradation by RNases and gut fluids, improved translocation across insect cuticle, and increased uptake in both plant and insect tissues. An overview of the polymers investigated for dsRNA delivery is provided in **Table 2**.

**Table 2:** Comparative overview of polymers used for dsRNA protection and delivery in RNAi-based pest control.

Polymer	Type	Key properties	Advantages	Limitations	References
PEI	Synthetic	Highly cationic; linear or branched (1.8–25 kDa).	Strong dsRNA binding, protection, and uptake; endosomal escape.	Cytotoxic at high MW or charge density.	(27, 30)
PEG-PEI	Modified synthetic	PEI grafted with PEG; reduced surface charge.	Better solubility and biocompatibility; lower toxicity.	Too much PEG reduces uptake efficiency.	(33, 35)
PAEMA	Synthetic	Cationic methacrylate with primary amines.	Efficient dsRNA complexation and stability; tunable MW.	High charge can cause mild cytotoxicity.	(36, 40)
PGUMA	Synthetic	Epoxide side chains allow chemical functionalization.	Versatile scaffold; stable and hydrophilic.	Complex synthesis; low biodegradability.	(38, 42)
Chitosan	Natural	Cationic polysaccharide; biodegradable and hydrophilic.	Eco-friendly; low toxicity; forms stable dsRNA complexes.	Lower transfection and pH-dependent solubility.	(44, 45)
Star Polycations (SPc)	Synthetic (branched)	Star-shaped polycationic nanostructure.	Strong dsRNA protection and delivery in plants/insects.	Synthesis complexity; possible toxicity.	(9, 49)
LDH (Bioclay)	Hybrid inorganic	Positively charged layered structure.	UV and nuclease protection; improved persistence and uptake.	Poor biodegradability; production variability	(46, 47)

Previous studies have demonstrated the successful application of synthetic polymer-based nanocarriers for RNAi delivery in various insect species, underscoring the versatility and adaptability of these materials in entomological biotechnology. For example, *Locusta migratoria* L. was effectively treated using poly(ethylene glycol)-polylysine(thiol) [PEG-PLys(SH)], a block copolymer with thiol functionalities that facilitate conjugation and cellular uptake (50). In another case, the lepidopteran pest *Spodoptera frugiperda* J. E. Smith responded positively to treatment with the cationic polymer poly-[N-(3-guanidinopropyl)methacrylamide] (pGPMA), which exploits guanidinium groups to enhance membrane penetration and nucleic acid binding (51). Similarly, in *Tribolium castaneum* Herbst, researchers successfully employed polyamidoamine dendrimer-functionalized carbon nanotubes (PAMAM-CNTs), leveraging the high surface area and multivalent binding capacity of dendrimers to improve RNAi efficacy (52). The Star Polycations carriers have been successfully applied in pests such as *Apolygus lucorum* Meyer-Dür (49) and *S. frugiperda* (53) showing improved gene silencing and pest control outcomes.

These examples collectively illustrate how the structural design and chemical functionality of polymeric carriers, such as charge density, hydrophilicity, and the presence of reactive groups, can significantly influence their performance in terms of delivery efficiency, cellular uptake, and target specificity. The observed species-specific responses further suggest that tailoring polymer architecture to the physiological and cellular characteristics of the target insect is essential. Consequently, the rational selection and engineering of polymeric nanocarriers represent a critical step in optimizing RNAi-based pest control strategies across diverse insect taxa.

### 2.2 Mechanisms and parameters of polyplex formation

The formation of stable complexes between dsRNA and cationic polymers is primarily driven by electrostatic interactions, where the positively charged polymer binds to the negatively charged phosphate backbone of dsRNA (54, 55). This electrostatic attraction facilitates the condensation of dsRNA into nanoscale polyplexes, which not only protect the RNA from enzymatic degradation but also enhance cellular uptake by promoting endocytosis (9, 56). The strength and stability of these complexes are influenced by several factors, including the polymer’s charge density, molecular weight, and architecture. A well-balanced interaction ensures efficient complexation without inducing excessive cytotoxicity, which is critical for the development of safe and effective RNAi-based delivery systems.

A critical parameter in this process is the N/P ratio, which reflects the balance between the cationic groups of the polymer and the anionic phosphate groups of the nucleic acid (9, 21). Optimizing the N/P ratio is essential for achieving efficient complexation: low N/P ratios may result in incomplete binding between the cationic polymer and the negatively charged RNA molecules, leading to unstable complexes that are prone to degradation by nucleases and inefficient cellular uptake (54, 57). This is because insufficient polymer content fails to fully neutralize and condense the RNA, leaving portions exposed and unprotected. On the other hand, excessively high N/P ratios can introduce several adverse effects. The surplus of cationic polymer increases the overall positive charge of the complexes, which can disrupt cellular membranes and induce cytotoxicity. Moreover, high polymer content may promote aggregation of the complexes, reducing their colloidal stability and hindering uniform distribution. These aggregates are less likely to be internalized efficiently by cells and may also interfere with endosomal escape, ultimately lowering transfection efficiency. Therefore, optimizing the N/P ratio is crucial to balance RNA protection, cellular compatibility, and delivery performance (54, 57). This balance is crucial for ensuring both the stability of the polyplex and the biocompatibility of the delivery system (54, 55).

The efficiency of complex formation between dsRNA and cationic polymers is commonly assessed using gel electrophoresis-based techniques, particularly gel retardation assays, zeta potential measurements and electrophoretic mobility shift assays (EMSA) (25, 56). These methods exploit the principle that nucleic acid–polymer complexes exhibit reduced electrophoretic mobility compared to free dsRNA due to changes in charge and size. In a gel retardation assay, increasing amounts of polymer are incubated with a fixed amount of dsRNA, and the resulting complexes are analyzed on an agarose gel. A retarded or absent migration band indicates successful complexation. Similarly, EMSA is used to detect shifts in nucleic acid mobility upon binding to proteins or polymers, providing insights into binding affinity, stoichiometry, and complex stability (58). Although EMSA is useful for visualizing complex formation, it has limitations such as low quantitative precision, potential disruption of weak interactions during electrophoresis, and inability to reflect intracellular behavior.

Additionally, the architecture of the polymer, whether linear, branched, or dendritic, plays a significant role in complexation efficiency and biological performance. The structural configuration directly influences the polymer’s ability to condense nucleic acids, form stable complexes, and facilitate cellular uptake (24, 29). Branched and dendritic polymers, such as branched polyethylenimine (bPEI) and dendrimers, typically exhibit higher charge density and a greater number of multivalent binding sites compared to their linear counterparts (59). This multivalency enhances electrostatic interactions with negatively charged nucleic acids, resulting in more compact and stable complexes that are better protected from enzymatic degradation. Moreover, the increased surface functionality of branched and dendritic architectures allows for improved interaction with cellular membranes, promoting endocytosis and intracellular delivery (24, 29, 30). These features contribute to higher transfection efficiency, especially in hard-to-transfect cell types (60). However, the same properties that enhance delivery can also increase cytotoxicity, necessitating careful optimization of polymer size, branching degree, and surface modifications. Recent studies have systematically compared the performance of linear and branched PEI in siRNA delivery systems. For instance, Bansal et al. (61) demonstrated that branched PEI-functionalized silica nanoparticles significantly improved siRNA retention and cellular uptake in glioblastoma models. Lungu et al. (29) provided a comparative analysis of the physicochemical properties of linear versus branched PEI, highlighting differences in complex stability and gene silencing efficiency. Ismail and Chou (24) further emphasized the role of dendritic structures in enhancing multivalent interactions and intracellular trafficking. Mohammadi et al. (30) reviewed the implications of PEI architecture on therapeutic outcomes, noting that dendritic and branched forms often outperform linear PEI in terms of delivery efficiency, albeit with higher toxicity risks.

To evaluate the physical and structural characteristics of dsRNA–polymer polyplexes, a diverse array of analytical tools is employed. Dynamic Light Scattering (DLS) is widely used to determine particle size distribution and zeta potential, which provide insights into colloidal stability, surface charge, and aggregation behavior of polyplexes (62). These parameters are crucial for predicting cellular uptake and biodistribution. Transmission Electron Microscopy (TEM) enables direct visualization of polyplex morphology at the nanoscale, revealing shape, compactness, and structural uniformity (63, 64). This method is particularly valuable for confirming successful complexation and assessing physical integrity. Gel-based methods, such as agarose gel electrophoresis, are commonly used to assess dsRNA integrity and binding efficiency (56). In addition, Electrophoretic Mobility Shift Assay (EMSA) is a sensitive technique used to study RNA–polymer interactions by detecting shifts in electrophoretic mobility when complexes form. EMSA can be applied to RNA–RNA, RNA–protein, or RNA–polymer complexes, and is particularly useful for evaluating binding specificity and affinity (58). Non-radioactive EMSA variants using fluorescent or biotin-labeled probes have become increasingly popular due to safety and sensitivity advantages.

Complementary techniques further enhance polyplex characterization:

- Atomic Force Microscopy (AFM) provides high-resolution topographical maps of polyplex surfaces, useful for evaluating surface roughness and mechanical properties (20, 65, 66).
- Fourier Transform Infrared Spectroscopy (FTIR) identifies chemical interactions between dsRNA and polymer functional groups, such as hydrogen bonding or electrostatic interactions (6, 67).
- UV–Visible Spectroscopy (UV–Vis) is used to quantify dsRNA concentration and monitor complex formation through absorbance shifts, especially in systems involving chromophoric polymers or dyes (68, 69).

Together, these factors must be carefully tuned to develop effective and safe dsRNA delivery systems tailored to specific biological applications, as the interplay between polymer architecture, charge density, and multivalency directly influences the stability, cellular uptake, and gene silencing efficiency of the resulting complexes. Optimizing these parameters is essential not only for maximizing delivery efficacy but also for minimizing cytotoxicity and off-target effects, thereby ensuring compatibility with the physiological environment of the target organism.

### 2.3 Stability of dsRNA–polymer polyplexes

The effectiveness of polymer-based dsRNA delivery systems is closely tied to the stability of polyplexes within biological environments. Insect midgut conditions, particularly pH and enzymatic activity, pose significant challenges to dsRNA integrity. For instance, dsRNA degradation is accelerated at pH levels between 6 and 10, while lower pH values (3–5) slow down this process (7, 62, 70). The midgut pH varies across insect orders: Coleoptera and Hemiptera typically exhibit mildly acidic conditions, whereas Orthoptera, Lepidoptera, Diptera, and Hymenoptera tend to have more alkaline environments (71–73). These physiological differences directly impact RNAi efficiency and necessitate the tuning of polymer properties to match the target organism’s internal conditions.

Published studies illustrate the diversity in polyplex stability, which significantly influences RNAi outcomes. Guanidinium-functionalized polymers, such as poly(oxanorbornene)imide (PONI-Guan), have demonstrated exceptional stability and functionality (74, 75). These polymers protect siRNA from degradation and facilitate direct cytosolic delivery, bypassing endosomal entrapment. Notably, PONI-Guan polyplexes retain gene knockdown efficacy even after lyophilization and reconstitution, achieving up to 80% *signal transducer and activator of transcription 3* (*STAT3*) knockdown and 70% inhibition of cell proliferation *in vitro* (74). In contrast, polyplexes formed with certain diblock copolymers or carriers with low charge density often exhibit poor stability under physiological or high-salt conditions, which compromises their *in vivo* applicability due to premature disassembly or insufficient cellular uptake (74). Comparative studies using fluorescence-based techniques, such as Förster Resonance Energy Transfer (FRET) and microscale thermophoresis (MST), have revealed that polyplexes with intermediate binding strength strike the optimal balance between extracellular protection and intracellular release. This balance is critical for effective RNAi, as overly strong binding can hinder cytosolic release, while weak binding may lead to premature degradation (74).

Polyplex stability is also highly sensitive to environmental factors such as pH, temperature, and ionic strength, which can significantly impact RNAi delivery efficiency. This is particularly evident in lepidopteran systems, where PAEMA-based polyplexes have shown poor stability, likely due to weak electrostatic interactions and lack of structural reinforcement (20). To overcome such limitations, chemical modifications can be introduced to enhance environmental resilience. For example, PGUMA, a guanidinium-functionalized polymer, has demonstrated improved stability at high pH due to its strong ionic interactions and ability to maintain compact polyplex structure even in basic conditions (76). Additionally, chemical crosslinking strategies, such as reducible click-linkages, can significantly improve polyplex stability under physiological conditions and reduce toxicity, offering a promising route for polymer modification (77).

In summary, ensuring the stability of dsRNA–polymer polyplexes is a critical step in developing effective RNAi delivery systems. This requires an integrated approach that considers environmental conditions, polymer architecture, and robust analytical characterization to support safe and targeted applications in insect pest management.

### 2.4 Biological performance and delivery efficiency

The efficacy of RNAi in insect pest management is strongly influenced by the chosen delivery method, the physiological barriers of the target species, and the design of the dsRNA–polymer complex. It is important to note that, despite advances in delivery technologies, RNAi effectiveness can vary widely across species and contexts, and successful gene knockdown is not always guaranteed. Delivery approaches such as oral feeding, injection, and topical application each offer distinct advantages and limitations depending on the biological and environmental context (20, 78–80). Oral delivery is particularly attractive for large-scale applications due to its non-invasive nature, but its success hinges on the stability of dsRNA in the insect gut and its ability to traverse the midgut epithelium. Injection bypasses digestive barriers and ensures direct access to the hemolymph, yet remains impractical for field deployment. Topical application, often enhanced by nanocarriers or surfactants, provides a compromise by enabling dsRNA absorption through the cuticle or ingestion during grooming.

A major challenge in oral and topical dsRNA delivery is its uptake across the insect midgut epithelium, which varies widely among insect orders. Coleopteran species, particularly members of the Chrysomelidae family such as the Colorado potato beetle (*Leptinotarsa decemlineata* Say) and the western corn rootworm (*Diabrotica virgifera virgifera* LeConte) exhibit high RNAi responsiveness, whereas many Lepidoptera, Diptera, and Hemiptera species often show limited or inconsistent sensitivity due to factors such as gut nucleases (dsRNases), reduced cellular uptake, and inefficient systemic spread of the RNAi signal (74, 79, 80). The midgut epithelium functions as a selective barrier, and successful RNAi requires dsRNA to resist enzymatic degradation and be internalized via endocytosis or channel-mediated transport. Environmental stressors, including UV radiation and microbial degradation, further compromise dsRNA integrity prior to ingestion (81, 82).

To overcome these barriers, polyplex-based delivery systems have been developed, wherein dsRNA is complexed with cationic polymers or nanoparticles to enhance stability, protect against nucleases, and facilitate cellular uptake (54, 55). The physicochemical properties of these polyplexes, particularly particle size, charge density, and surface chemistry, play a critical role in determining RNAi efficacy across insect species (9, 21, 29). Polyplexes with intermediate binding strength often achieve an optimal balance between extracellular protection and intracellular release, ensuring that dsRNA remains intact during transit but is still accessible to the RNAi machinery once inside the cell. For instance, a comparative study in *Aedes aegypti* L. evaluated chitosan, carbon quantum dots, and silica nanoparticles for dsRNA delivery, showing that each system offered distinct advantages in stability, cellular uptake, and RNAi efficiency (83). This highlights the importance of tailoring nano-delivery strategies to the target species and biological context. Similarly, studies in orthopteran pests such as *Schistocerca gregaria* Forsskål and *Melanoplus sanguinipes* Fabricius demonstrated that nanoparticle-mediated dsRNA delivery improved stability and cellular uptake, but RNAi efficiency remained species-specific, highlighting the need to tailor delivery strategies to target insect physiology (84, 85).

However, even with optimized polyplexes, some species such as *S. frugiperda* and other lepidopterans or hemipterans may fail to show effective gene knockdown due to rapid dsRNA degradation, low endocytic activity, or other physiological barriers (16, 86–89). These outcomes illustrate that stability and delivery alone do not guarantee functional RNAi across all insect taxa.

In conclusion, the success of RNAi-based pest control strategies depends on a nuanced understanding of delivery routes, epithelial uptake mechanisms, and polyplex design. Integrating species-specific physiological data with polymer chemistry is essential for developing robust and scalable RNAi platforms suitable for diverse insect taxa and environmental conditions. It is important to note that even with optimized delivery systems, physiological and environmental barriers can limit RNAi effectiveness, indicating that stability and cellular uptake alone may not always ensure successful gene knockdown.

### 2.5 Design criteria for polymer selection

The development of effective polymer-based dsRNA delivery systems for insect pest management hinges on a careful balance of multiple design parameters. Prior studies have consistently emphasized four core criteria:

- Stability: Polymers must protect dsRNA from enzymatic degradation in the insect midgut and from environmental stressors such as UV radiation and microbial activity (21).
- Complexation Efficiency: Strong electrostatic interactions between cationic polymers and the negatively charged dsRNA backbone are essential for forming stable polyplexes that facilitate cellular uptake (9, 90).
- Toxicity: While high charge density improves delivery, it often correlates with increased cytotoxicity. Thus, polymer architecture and molecular weight must be optimized to minimize adverse effects to nontarget organisms (9, 21, 78).
- Biodegradability: Environmentally sustainable pest control requires polymers that degrade safely without accumulating in ecosystems or harming non-target organisms (78).

Despite these advances, several critical gaps remain in current research. One major limitation is the lack of standardized protocols for assessing polyplex stability, particularly under biologically relevant conditions such as insect midgut fluids or field environments (78). Additionally, comparative data across insect species are scarce, making it difficult to generalize findings or predict RNAi efficacy in non-model organisms (21). Finally, environmental safety assessments of polymer carriers are often underdeveloped, despite their importance for regulatory approval and ecological sustainability (78).

Addressing these gaps will be essential for advancing RNAi-based pest control technologies. Future research should prioritize cross-species comparative studies, harmonized testing protocols, and comprehensive environmental risk assessments to ensure safe and scalable deployment of polymer-based dsRNA delivery systems.

## 3 Discussion

Polymer-based dsRNA delivery systems have emerged as a promising tool in insect pest management, yet their practical implementation remains constrained by several unresolved challenges. While numerous studies have demonstrated the potential of cationic polymers to stabilize dsRNA and enhance cellular uptake, the current body of knowledge is fragmented and lacks methodological coherence, limiting reproducibility and field applicability (11, 91).

A key consideration in current research is the variation in experimental parameters, which can influence the interpretation and comparability of results. For example, studies differ in their use of N/P ratios, ranging from 1:1 to over 30:1, depending on the polymer type and target organism (91). These ratios affect both complexation efficiency and cytotoxicity, yet are rarely standardized across studies. dsRNA lengths vary widely—from short siRNA fragments (~21 bp) to long dsRNA molecules (>500 bp)—with intermediate-length strands (e.g., 100–300 bp) often offering a balance between silencing potency and stability. While longer dsRNAs can enhance RNAi efficacy by serving as Dicer substrates, they are also more susceptible to degradation, particularly in insect systems with high nuclease activity (92). Buffer systems such as phosphate-buffered saline (PBS), 4-(2-hydroxyethyl)-1-piperazineethanesulfonic acid (HEPES), or tris(hydroxymethyl)aminomethane (Tris) differ in ionic strength and pH, which can influence polyplex formation and stability, yet few studies compare their effects systematically (50). While these differences reflect the diversity of biological models and experimental goals, they also underscore the need for more harmonized approaches to support reproducibility and facilitate cross-study comparisons in polymer-based RNAi delivery research.

Moreover, while many studies report promising *in vitro* stability and transfection efficiency, these results do not always translate into comparable *in vivo* efficacy. For instance, Bai et al. (93) showed that dsRNA remained stable under various *in vitro* conditions, including freeze-thaw cycles, but RNAi efficiency in *T. castaneum* did not consistently reflect this stability. This suggests that current *in vitro* models may not fully capture the physiological complexity of insect systems, particularly the midgut environment and immune responses (94). Several studies in both lepidopteran and hemipteran insects have been demonstrate that even when dsRNA is efficiently protected and forms stable complexes with polymeric carriers, it often still fails to produce measurable gene knockdown. This limited RNAi responsiveness has been attributed to several potent physiological barriers, including high gut dsRNase activity, which rapidly degrades dsRNA before uptake (87, 89), low or inefficient endocytic pathways that restrict internalization into midgut epithelial cells (86), and rapid gut transit, which shortens the time window for dsRNA absorption (16, 95). Taken together, these studies clearly show that dsRNA stability, whether achieved through polymeric carriers or other nanomaterials, is not in itself sufficient to ensure functional RNA interference, highlighting the importance of accounting for strong species-specific physiological constraints when designing delivery systems.

Another limitation is the species bias in RNAi research, where early studies focused heavily on two highly responsive coleopteran species—*L. decemlineata* and *D. virgifera virgifera*. This led to a misleading perception that Coleoptera are broadly RNAi-sensitive, although most examined species show low responsiveness, especially at realistic dsRNA doses (80). As a result, delivery systems have often been optimized for these beetle models, while many RNAi-sensitive lepidopteran and hemipteran species remain underrepresented in delivery-focused studies (42, 96). In contrast, many other economically and ecologically important insects remain underrepresented in delivery-focused studies. These include lepidopteran pests such as *S. frugiperda*, *Helicoverpa armigera* Hübner, *Plutella xylostella* L, and *Trichoplusia ni* Hübner, as well as hemipteran pests like aphids (*Acyrthosiphon pisum* Harris, *Myzus persicae* Sulzer), whiteflies (*Bemisia tabaci* Gennadius), and planthoppers (*Nilaparvata lugens* Stål), which are known for their challenging gut environments and low RNAi responsiveness (15, 16, 97, 98). Without comparative data spanning these taxa, it remains difficult to generalize polymer performance or accurately predict RNAi outcomes in non-model insects. To move the field forward, future research must address several critical needs:

- Tailored polymer design that considers taxon-specific factors such as gut physiology, dsRNase activity, and epithelial uptake mechanisms, which vary significantly across insect taxa (75, 79).
- Stronger correlation between *in vitro* and *in vivo* performance, supported by predictive assays that better simulate insect midgut conditions and biological complexity (50, 79).
- Comprehensive environmental safety assessments, including studies on degradation kinetics, non-target organism effects, and ecological persistence, which are essential for regulatory approval and sustainable field application (6, 78, 99).

Finally, the continued advancement of RNAi delivery systems will rely on interdisciplinary collaboration. By integrating expertise from polymer chemistry, entomology, molecular biology, and environmental science, researchers can develop delivery platforms that are not only efficient and species-specific, but also safe, scalable, and environmentally sustainable (11, 99).

## 4 Conclusions

Polymer-based dsRNA delivery systems hold significant promise for advancing insect pest management through RNAi, yet their practical deployment is hindered by methodological inconsistencies, species-specific limitations, and gaps in translational efficacy. The current literature reveals a fragmented landscape, where variations in N/P ratios, dsRNA lengths, buffer systems, and model organisms complicate cross-study comparisons and reproducibility. Bridging the gap between *in vitro* success and *in vivo* performance requires more physiologically relevant testing models and predictive assays that reflect the complexity of insect biology. Expanding research to include a broader range of insect taxa is essential for developing delivery platforms that are effective across diverse orders, particularly those with variable RNAi responsiveness.

To move the field forward, future efforts must prioritize tailored polymer design, standardized methodologies, and robust environmental safety assessments. Interdisciplinary collaboration will be key to developing RNAi delivery systems that are not only effective and species-specific but also scalable and ecologically responsible. By addressing these challenges, polymer-based RNAi technologies can evolve into reliable tools for sustainable pest control in agricultural ecosystems.
